# Fabrication and Characterization of a Novel Solid Nano-Dispersion of Emamectin Benzoate with High Dispersibility and Wettability

**DOI:** 10.3390/nano15070495

**Published:** 2025-03-26

**Authors:** Ying Li, Qing Wang, Junqian Pan, Xiang Zhao, Jinghui Zhan, Xinglong Xu, Meng Zhang, Chunxin Wang, Haixin Cui

**Affiliations:** 1 College of Resources and Environment, Shanxi Agricultural University, Taigu 030800, China; z20223452@stu.sxau.edu.cn (Y.L.); 20233523@stu.sxau.edu.cn (Q.W.); 2 Institute of Environment and Sustainable Development in Agriculture, Chinese Academy of Agricultural Sciences, Beijing 100081, China; panjunqian@caas.cn (J.P.); zhaoxiang@caas.cn (X.Z.); jh_zhan9679@163.com (J.Z.); 82101235293@caas.cn (X.X.); 82101225293@caas.cn (M.Z.)

**Keywords:** nanopesticides, emamectin benzoate, dispersibility, wettability, solid nano-dispersion

## Abstract

Pesticides, as an indispensable component in agricultural production, play a crucial role in ensuring global food security. However, the low efficiency of pesticide utilization remains a significant challenge. The key method of improving the effective utilization rate of pesticides is mainly to enhance the affinity between pesticides and leaf surfaces while improving their deposition and adhesion properties. In this study, we utilized PEG 4000 as a carrier and emulsifier 600 and emulsifiers 700 as surfactants to prepare solid nano-dispersion of emamectin benzoate (SND-EB) by the melting method. SND-EB particles were spherical with an average diameter of 17 nm, a loading capacity of up to 50%, and excellent dispersibility. Contact angle and bouncing behavior tests on cabbage and pepper leaves demonstrated that SND-EB had superior wetting properties and spreading capabilities. Surface tension and leaf retention measurements further confirmed that SND-EB possessed excellent adhesion and leaf affinity. The SND-EB showed a 1.8-fold increase in biological activity against *Spodoptera exigua* compared to commercial emamectin benzoate water-dispersible granule (WDG-EB). In addition, the fabricated nanoparticles exerted no toxic effect on HepG2 cells. These results demonstrated that a 50% content of SND-EB exhibited excellent water dispersity, wettability, and insecticidal activity, providing a novel and efficient strategy for pest control.

## 1. Introduction

With the global population increase and continuous reduction in agricultural arable land, pesticides have become indispensable agricultural chemicals to meet the growing demand for food and ensure stable and high yields of crops [1]. Studies project that by 2050, global food demand will rise by 70% to 100%, and pesticides play an important role in increasing agricultural production and preventing pests and diseases [2,3]. However, determining how to safely use pesticides is crucial for controlling pesticide residue pollution and ensuring the quality of agricultural products.However, pesticides can also be a double-edged sword; underutilized or improperly managed pesticides can enter the environment, causing pollution to soil, surface water, and aquatic organisms [4,5,6], as well as posing threats to human health [7,8,9]. In this context, the rapid development of nanotechnology offers new methods for promoting sustainable agricultural development.

With the cross-integration of nanoscience and agriculture, nanotechnology has emerged as a pivotal tool for accelerating cutting-edge innovation in agriculture and fostering sustainable agricultural development [10]. Over the past few decades, the application of nanotechnology in plant and agricultural sciences has garnered significant attention [11,12,13]. In 2019, nanopesticide was recognized by the International Union of Pure and Applied Chemistry (IUPAC) as one of the foremost emerging chemical technologies poised to transform the world [14]. Current formulations of nanopesticides encompass nanoemulsions, nanosuspensions, mesoporous nanoparticles, and solid nanopowder [15,16,17]. The advantages of nanopesticides include (1) enhancing pesticide adhesion on leaf surfaces, thereby improving biological activity and efficacy [18,19,20], and (2) facilitating the construction of long-lasting, controlled-release nano-carrier systems, and then improving the precision, targeting, and efficiency of pesticide delivery [21,22,23,24,25]. However, challenges remain, such as low pesticide utilization rates, poor solubility, easy drift of pesticide-loaded particles, and environmental and health risks posed by organic solvents.

Solid dispersion (SD) is a solid dispersion system formed by highly dispersing insoluble drugs in a molecular, colloidal, microcrystalline, or amorphous state in solid materials. It is widely regarded as an effective strategy for improving the solubility of insoluble drugs due to its simplicity, speed, and maturity [26]. Solid dispersion can change crystal structure to transform drugs into amorphous state, increase Gibbs free energy, and improve the solubility of active ingredients [27]. In addition to improving the crystal structure, solid dispersion technology can improve the dispersibility of drugs, reduce the possibility of drug accumulation and precipitation, and thus improve the solubility and dissolution of active ingredients in traditional Chinese medicine in a medium. In addition, the active ingredients in drugs can also form water-soluble complexes with carriers to achieve solubility enhancement [28,29]. Cui et al. prepared a highly efficient solid dispersion of lambda-cyhalothrin using the melt emulsion solidification method, with an average particle size of 21.7 nm, significantly improving the water solubility of lambda-cyhalothrin. The particle size could be maintained below 85 nm under refrigeration and thermal storage conditions [30]. Huang et al. used solid dispersion technology to prepare methomyl benzoate (EM). The EM-PVP-K30 wettable powder prepared had a water solubility 37.5 times that of EM, greatly improving the sustained-release effect and insecticidal activity [31].

Emamectin benzoate, a macrocyclic lactone biological insecticide, is a novel semisynthetic derivative antibiotic produced from the fermentation product of avermectin B1. However, its poor water solubility and rapid degradation characteristics significantly impede its application in agricultural production. With the advancement of technology, various formulations of emamectin benzoate have been developed [32,33,34]. At present, the main dosage forms of emamectin benzoate include microemulsion, emulsion, suspension, water-dispersible granule, water emulsion, microcapsule suspension, suspension seed coating agent, etc. Solid powders such as powders, granules, and wettable powders have coarse drug-carrying ions, which are prone to drift and scatter into the environment, resulting in low pesticide release rates, low pesticide loading per unit area, and low effective pesticide utilization rates. Although the emulsion is diluted with water and easy to spray, it contains a large number of organic solvents, which can easily cause soil, air, and water pollution. In addition, most of the surfactants used in microemulsions are solubilizers, which can easily penetrate into farmland and water sources, and their toxicity cannot be ignored. The preparation of nanopesticides can effectively reduce the use of organic solvents, improve pesticide efficacy, and prolong shelf life; Solid dispersions can increase the solubility of pesticides, improve crystal structure, and enhance bioavailability. Therefore, combining nanotechnology with solid dispersion technology for the preparation of methomyl salts can bring many advantages. However, there are limited reports on nano-solid dispersions [35].

In this study, an emamectin benzoate solid nano-dispersion (SND-EB) was prepared using PEG 4000 as a carrier via the melting method. The microscopic morphology and physiochemical properties of SND-EB were characterized using scanning electron microscopy (SEM), transmission electron microscopy (TEM), Fourier transform infrared spectroscopy (FT-IR), X-ray diffraction (XRD). The sustained-release properties of the solid nano-dispersion, its wetting and adhesion properties on cabbage and pepper leaves, and its biological efficacy against *Spodoptera exigua* were thoroughly investigated. In addition, the toxicity of the solid nano-dispersion on HepG2 cells was also studied. The pesticide content of the SND-EB prepared in this study was 50%, while the amounts of emulsifier and carrier were greatly reduced. On the basis of maintaining excellent wetting and dispersion performance, the SND-EB greatly reduced environmental pollution and improved the utilization rate of pesticides.

## 2. Materials and Methods

### 2.1. Materials

Emamectin benzoate technical material (TC, 95%, *w*/*w*) and standard (97.1%, *w*/*w*) were purchased from Hebei Weiyuan Biochemical Co., Ltd. (Shijiazhuang, China) and AccuStandard (Shenyang, China), respectively. Emamectin benzoate water-dispersible granules (WDGs, 5%, *w*/*w*) were purchased from Shanghai Yuelian Biotechnology Co., Ltd. (Shanghai, China). Methanol and polyethylene glycol 4000 were purchased from Shanghai McLean Biochemical Technology Co., Ltd. (Shanghai, China). Styrene-based phenol polyoxyethylene ether (emulsifier 600) and alkylphenol formaldehyde resin polyoxyethylene ether (emulsifier 700) were purchased from Jiangsu Haian Petrochemical Plant (Nantong, China). Chromatographic-grade methanol, acetonitrile, and triethylamine were purchased from Thermo Fisher Scientific Co., Ltd. (Shanghai, China). All the chemicals were used as received. Milli-Q water (18 MΩ cm, TOC ≤ 4 ppb) was used in all analytical experiments.

### 2.2. Preparation of SND-EB

Firstly, the weighed emulsifier 600, emulsifier 700, and emamectin benzoate were added into the same beaker (emulsifier/EB = 1:2. emulsifier 600/emulsifier 700 = 3:2), and then 150 mL of methanol was poured into the mixture to form a transparent solution A under heating and stirring conditions. Secondly, the weighed polyethylene glycol 4000 was transferred to a round-bottom flask and heated and stirred in a water bath until transparent. Thirdly, Solution A was dropped into a round-bottom flask slowly using a separatory funnel. Simultaneously, the mixture was continuously stirred and heated for half an hour, and then the solution was evaporated by rotary evaporation. Finally, the evaporated product was rapidly cooled and crushed using a pulverizer to obtain SND-EB.

### 2.3. Determination of SND-EB Content

The pesticide content in the formulation was analyzed by high-performance liquid chromatography (HPLC, 1260 Infinity, Agilent, Santa Clara, CA, USA) using a C18 column (5 μm, 4.6 mm × 250 mm, Zorbax Eclipse Plus C18, Agilent, Santa Clara, CA, USA) at 30 °C. The mobile phase was composed of acetonitrile, methanol, and 0.1% (*v*/*v*) triethylamine aqueous solution (50:40:10, *v*/*v*/*v*). The flow rate was 1.0 mL min^−1^, and the UV detector wavelength was 245 nm.

### 2.4. Morphology Characterization

The morphology of SND-EB was characterized by scanning electron microscopy (SEM, JSM-7401F, JEOL, Tokyo, Japan). Specifically, 0.1 g of the as-prepared sample was put in a centrifuge tube containing 10 mL of deionized water. After 5 min of ultrasonic treatment, the sample was dropped onto a silicon wafer and allowed to dry naturally. Platinum was sprayed onto the sample using a sputtering coating machine (ETD-800, Beijing Weitu Technology Development Co., Ltd., Beijing, China).

The morphology of SND-EB was characterized by transmission electron microscopy (TEM, HT7700, Hitachi, Tokyo, Japan). Specifically, 0.1 g of the sample was taken out and heated in a centrifuge tube containing 10 mL of deionized water. After 5 min of ultrasonic treatment, the sample was dropped onto a copper mesh.

### 2.5. Fourier Transform Infrared Spectroscopy (FT-IR)

The Fourier transform infrared spectrum of the sample was obtained using a Fourier transform infrared spectrometer. The sample was pressed into tablets for FT-IR analysis. Each sample was scanned within the spectrum of 400 to 4000 cm^−1^.

### 2.6. X-Ray Diffraction Analysis

XRD (Rigaku SmartLab SE, Tokyo, Japan) was used to characterize the crystal morphology of SND-EB and EB. The sample was dried before measurement. Cu Kɑ radiation was generated at 40 kV voltage and 40 mA current, and the sample was analyzed within the 2θ range of 5–90.

### 2.7. Particle Size and Zeta Potential Measurements

The hydrodynamic particle sizes, polydispersity index (PDI), and zeta potentials of SND-EB were measured at room temperature by a Zetasizer Nano ZS 90 (Malvern, Worcestershire, UK). All of the particle sizes and PDI were tested in triplicate and recorded as mean ± standard deviation (S.D).

### 2.8. Storage Stability Test

After being sealed, the samples were stored at temperatures of 4 °C, 25 °C, and 54 °C for 14 d. During this period, samples were taken on 3, 5, 7, and 14 d. The particle size, PDI, and zeta potential were measured after the collected samples were dispersed in water. In addition, stability was evaluated by measuring pesticide content using the liquid phase.

### 2.9. Static Contact Angle

The static contact angles of SND-EB, WDG-EB, and water on cabbage and pepper leaves were measured using a contact angle meter (DSA-100, Krüss GmbH, Hamburg, Germany). Fresh cabbage and pepper leaves were washed with water and dried naturally. They were then attached to a glass slide to measure the static contact angle. Finally, the contact angle of the droplets was determined using the five-point fitting method. The experiment was repeated three times, and the average was taken.

### 2.10. Dynamic Contact Angle

The dynamic contact angles of SND-EB, WDG-EB, and water on cabbage and pepper leaves were measured using a contact angle apparatus (DSA-100, Krüss GmbH, Hamburg, Germany). Fresh cabbage leaves from the greenhouse were selected, and any dust on the leaf surface was carefully removed without damaging the foliar structure. After the leaves were naturally air-dried, they were adhered to glass slides for use. The sample solution was dropped onto the leaves, and the state of the droplets was continuously observed and recorded for 180 s. This was repeated three times for each sample.

### 2.11. Bouncing Behavior

The impact processes of SND-EB, WDG-EB, and water droplets on cabbage and pepper leaves were recorded by a high-speed camera (FASTCAM Mini UX100, Photron, Tokyo, Japan) from the oblique and profile views with 2000 frames per second. The droplets fell perpendicular to the cabbage leaves at the same initial height (20 cm), ensuring that each droplet had the same impact velocity. The impact velocity of all droplets was −2.0 ms^−1^. The experiment was repeated three times.

### 2.12. Leaf Retention

Dry pepper leaves were weighed and immersed in the sample solutions for 30 s and then lifted vertically with tweezers. The leaf was weighed again after the sample solution stopped dripping from it, and the liquid-holding capacity (LHC) per unit area was calculated. Here, S is the area of the cut pepper leaves, and m_2_ (g) and m_1_ (g) are the masses of the leaves before and after immersing, respectively.

(1) $$LHC=\frac{m_{1}-m_{2}}{2S}$$

### 2.13. Measurement of Surface Tension

The dilutions of the samples and control samples were measured using a surface tension meter (BP100, Krüss GmbH, Hamburg, Germany). After the instrument was adjusted to a horizontal position, it was calibrated with pure water before testing. Then, the measuring ring and the glass dish containing the sample were cleaned with anhydrous ethanol and dried with a hair dryer. Finally, the surface tension value of the sample was measured three times. This cleaning step was repeated for each sample before proceeding to the next sample measurement.

### 2.14. Experiment Study of EB Release from SND-EB

A dialysis bag with a molecular weight of 10,000 was cut into 10 cm lengths and was boiled at 500 °C for 10 min. Then, the dialysis bag was naturally cooled to room temperature and soaked in pure water for later use. Then, 20 mg SND-EB and 13.3 mg EB were dissolved in 3 mL of release medium and placed in a dialysis bag. Then, the dialysis bag was placed into 100 mL of release medium containing 50% ethanol. The samples were stirred at 100 r/min on a shaker. Then, 1 mL of solution was taken out at 0.5 h, 1 h, 3 h, 6 h, 10 h, 24 h, 36 h, 48 h, 72 h, 96 h, and 120 h, respectively. Then, 1 mL of release medium was added to maintain the total volume of the solution. The concentration of emamectin benzoate was measured by liquid chromatography, and the cumulative release amount was calculated at different time points.

### 2.15. Biological Testing Experiment

The insecticidal activity of the SND-EB was tested using the poison bait method. Firstly, the solutions of SND-EB and WDG-EB were diluted to obtain five different concentrations (0.25, 0.125, 0.0625, 0.03125, 0.015625 ppm). Pakchoi (*Brassica chinensis* L.) leaves were selected as model test leaves and were cut into 3 × 3 cm^2^ squares. The cut leaves were soaked in pesticide dilution solution for 1 min and then naturally air dried and transferred to a culture dish. Ten third-instar beet armyworms (*Spodoptera exigua*) were placed in culture dishes, and the mortality rate of cabbage armyworms was calculated using the Abbott formula and LC_50_ after 24 h. The toxicity regression equation was calculated using probability analysis. Each experiment was repeated 3 times to eliminate other interfering factors.

### 2.16. Cell Viability Experiment

HepG2 cells were incubated in Dulbecco’s modified Eagle medium containing fetal bovine serum. Then, the culture medium was placed in a 37 °C and 5% carbon dioxide incubator. The cytotoxicity of Nano EB and WDG-EB was evaluated separately. The diluent was prepared according to the design concentration, and the control group was an equal volume of serum-containing culture medium. The absorption values at 490 nm in each well were measured using an enzyme-linked immunosorbent assay (ELISA) reader. Blank cells with only culture medium added were used as control wells, and equal volumes of culture medium, MTT, and DMSO were added as zeroing wells. Cell viability = [A490 (sample)/A490 (control)].

## 3. Results

### 3.1. Particle Size Distribution and Morphology of SND-EB

**Figure 1:**
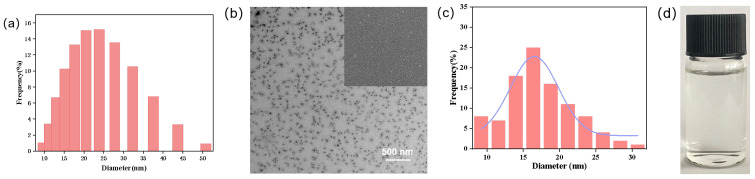
(**a**) DLS image, (**b**) SEM image, (**c**) statistical particle size based on SEM image, (**d**) the state of diluent.

The samples were dispersed in deionized water for the measurements. As shown in Figure 1a, the mean particle size and PDI were 20 nm and 0.181, respectively. A PDI value of less than 0.3 suggested a fairly narrow size distribution and could be used to predict the system stability. As shown in Figure 1b, the morphology of the nanoparticles was almost spherical. As shown in Figure 1c, the mean particle size based on SEM images was 17 nm, which was consistent with the hydrodynamic size determined by DLS. In Figure 1d, the diluted solution of SND-EB appears transparent.

### 3.2. Structural Characterization of SND-EB

**Figure 2:**
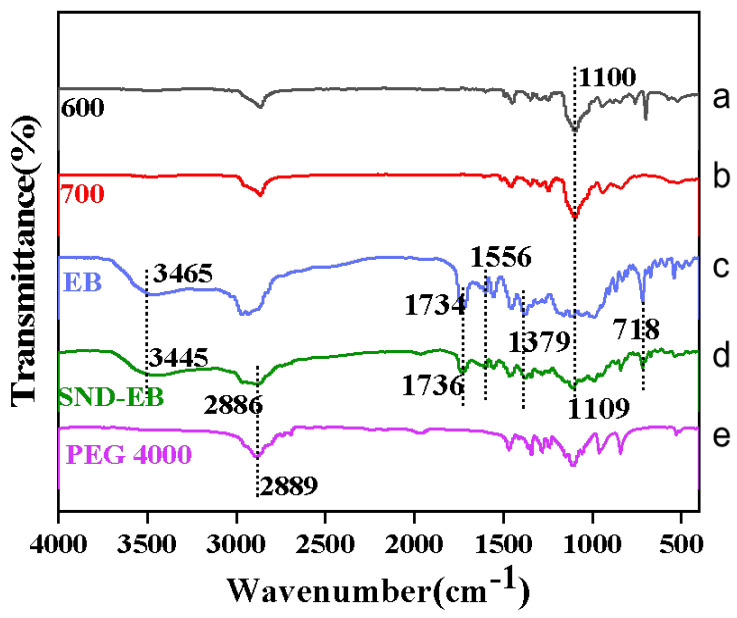
FT-IR spectra of emulsifier 600, emulsifier 700, EB, SND-EB, and PEG 4000. a: Fourier transform infrared spectroscopy of emulsifier 600; b: Fourier transform infrared spectroscopy of emulsifier 700; c: Fourier transform infrared spectroscopy of EB; d: Fourier transform infrared spectroscopy of SND-EB; e: Fourier transform infrared spectroscopy of PEG 4000.

As shown in Figure 2, EB exhibited a characteristic peak at 3465 cm^−1^, which is the stretching vibration absorption of ammonium groups, while the same absorption peak appeared at 3445 cm^−1^ in SND-EB. Compared with EB, except for the characteristic peak at 3445 cm^−1^, the other absorption peak in the spectrum of SND-EB remained basically unchanged. In addition, some characteristic peaks of SND-EB belonged to adjuvants and carriers. Emulsifier 600 and emulsifier 700 exhibited characteristic peaks at 1096 cm^−1^ (-C-O ether bond strong absorption peak) and 1097 cm^−1^ (-C-O ether bond strong absorption peak), respectively. The same absorption peak appeared at 1100 cm^−1^ on the SND-EB spectrum. PEG 4000 exhibited a characteristic peak (-CH_2_ stretching vibration) at 2889 cm^−1^, and the same absorption peak appeared in SND-EB at 2886 cm^−1^ [36,37,38]. The above conclusion indicated that SND-EB still maintains the structure of methomyl without chemical or structural changes.

**Figure 3:**
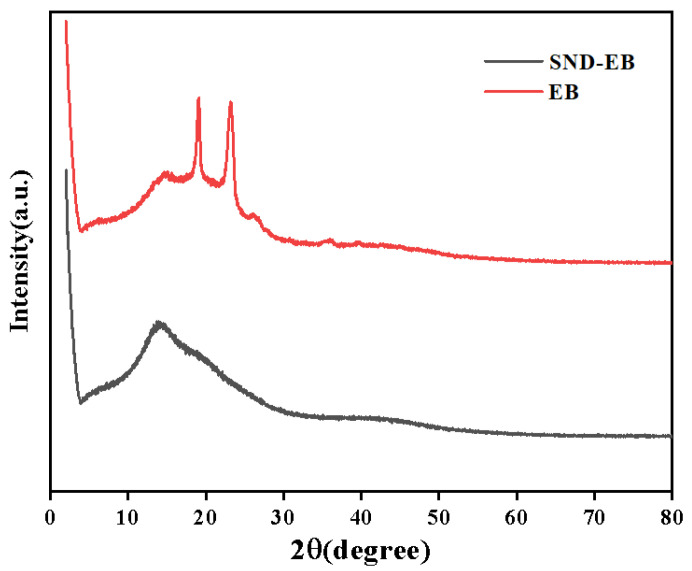
XRD of EB and SND-EB. The red line represents the X-ray diffraction pattern of EB, and the black line represents the X-ray diffraction pattern of SND-EB.

The spectra of emamectin benzoate and the prepared SND-EB are shown in Figure 3. The emamectin benzoate technical drug itself was a crystal and had sharp characteristic peaks in XRD. However, the XRD spectrum of the newly prepared SND-EB showed a wide peak, indicating that the SND-EB was amorphous. The crystal form directly affected the water dispersibility of the nano-dispersion. During the preparation process, the crystal state of the emamectin benzoate changed from crystal to amorphous, and the water dispersibility and solubility of the SND-EB were improved.

### 3.3. Stability Analysis

**Figure 4:**
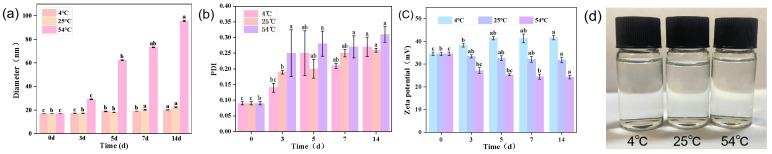
(**a**) Particle size stability of SND-EB at 4 °C, 25 °C, and 54 °C; (**b**) PDI stability of SND-EB at 4 °C, 25 °C, and 54 °C; (**c**) zeta stability of SND-EB at 4 °C, 25 °C, and 54 °C; (**d**) diluent state under conditions of 4 °C, 25 °C, and 54 °C. The different letters in the figure indicate significant differences when *p* < 0.05.

The stability of SND-EB was verified after storage at 4 °C, 25 °C, and 54 °C for 14 d. As shown in Figure 4a,b, the average particle size and PDI of SND-EB did not change significantly at 4 °C and 25 °C for 14 d. The particle size increased from 17 nm to 22 nm, and the PDI was maintained under 0.3 after storage at 0 °C for 7 d. Under low-temperature storage conditions, the Brownian motion between particles weakens, and the degree of aggregation between particles decreases, resulting in relatively small changes in particle size. In addition, the structure of emulsifier 700 could provide steric hindrance, preventing particle aggregation and crystal growth. As shown in Figure 4c the zeta potential of SND-EB can still maintain above 30 mV at 4 °C/25 °C for 14 days, indicating good stability. In addition, the stored sample could still remain in a clear and transparent lotion state when diluted with water in Figure 4d. The sample exhibited physical stability under refrigeration and room temperature conditions.

Under the storage condition of 54 °C for 14 d, the water-dispersed particle size of SND-EB gradually increased to 62.24 nm after 5 d and increased to 94.64 nm after 14 d. The melting point range of PEG 4000 was 53–58 °C, and SND-EB may agglomerate because of the melting of PEG 4000, which caused an increase in the particle size. PEG 4000 melted and destroyed the structure of SND-EB after 14 d at 54 °C, which weakened the encapsulation of SND-EB by PEG 4000. The particles of the pesticide active ingredient may undergo Ostwald ripening, leading to particle adhesion and an increase in particle size [39,40,41]. However, the sample could still remain in a clear and transparent lotion state when diluted with water, and there was no sedimentation or stratification of the diluent after 54 °C for 14 days. In summary, this meant that the sample could still maintain good stability. The zeta potential of SND-EB decreased from 34 mV to 24 mV at 54 °C for 14 days. As the temperature increased, the ion mobility increases, the particle diffusion rate in the solution increases, and the double layer is compressed, resulting in a decrease in zeta potential. In addition, the double-layer structure on the electrode surface was disrupted at high temperatures, leading to a decrease in potential. Owing to the combined electrostatic and steric stabilizer, a zeta potential of ±20 mV would be acceptable.

**Figure 5:**
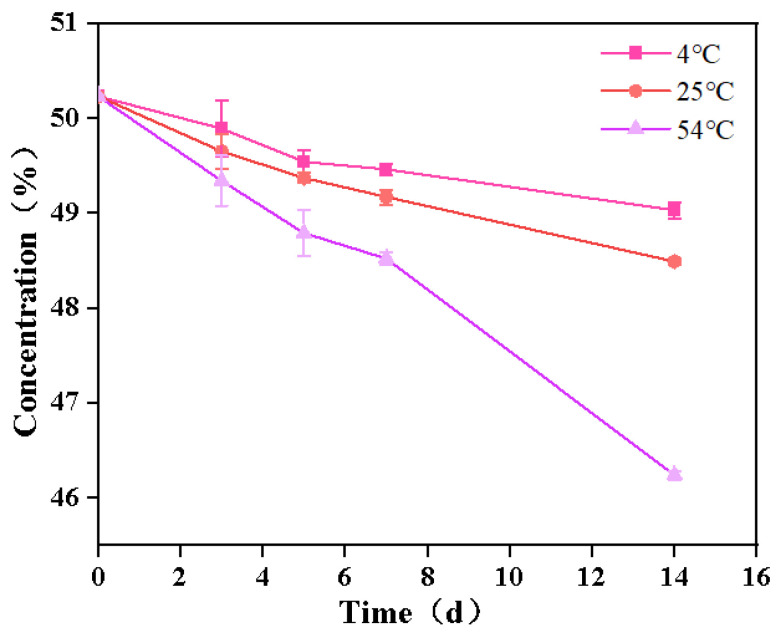
Concentration changes of SND-EB at 4 °C, 25 °C, and 54 °C for 14 days.

Pesticides may decompose during storage or transportation due to external environmental factors, leading to a decrease in pesticide content. Therefore, the content changes of SND-EB were studied after storage at 4 °C, 25 °C, and 54 °C for 14 d. In Figure 5, the concentration of SND-EB changed little, decreasing from 50% to 49.03% at 4 °C and from 50% to 48.49% at 25 °C. The above results indicated that SND-EB had excellent storage stability at 4 °C and 25 °C. Polymers could serve as inhibitors of drug crystallization. PEG 4000 could form hydrogen bonds with pesticide raw materials, which could to some extent maintain the stability of drugs. The concentration of SND-EB decreased from 50% to 46% at 54 °C for 14 d, which could meet the stability storage requirements of pesticide formulations.

### 3.4. Wettability, Spreading, and Retention

The contact angle is one of the important indicators for evaluating the wetting performance of pesticide droplets on plant leaf surfaces, which can reflect the adhesion of the pesticide solution on the leaf surface [42]. In order to investigate the wetting properties of solid nano-dispersions, the static contact angle, dynamic contact angle, and droplet impact behavior of water, SND-EB, and WDG-EB on hydrophilic leaves (pepper) and hydrophobic leaves (cabbage) were evaluated.

**Figure 6:**
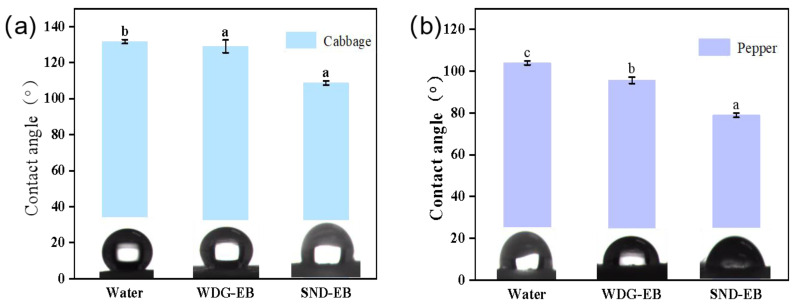
(**a**) Static contact angles of water, WDG-EB, and SND-EB on cabbage leaves; (**b**) static contact angles of water, WDG-EB, and SND-EB on pepper leaves; Different letters in the plot indicate significant difference at *p* < 0.05.

The better the wetting performance of the solution, the smaller the contact angle on the leaf surface [43,44]. Due to the wax layer structure on the surface of cabbage leaves, their hydrophobicity is significantly stronger than that of chili leaves. As shown in Figure 6, the static contact angle of deionized water on cabbage leaves was 131°, while the contact angles of SND-EB and WDG-EB on cabbage leaves were 108° and 129°, respectively. The contact angle of deionized water on pepper leaves was 104°, while the contact angles of SND-EB and WDG-EB on pepper leaves were 79° and 95°, respectively. Compared to WDG-EB, SND-EB droplets had a smaller contact angle and better foliar spreading, resulting in better diffusion and wetting performance of droplets on the target crop surface.

**Figure 7:**
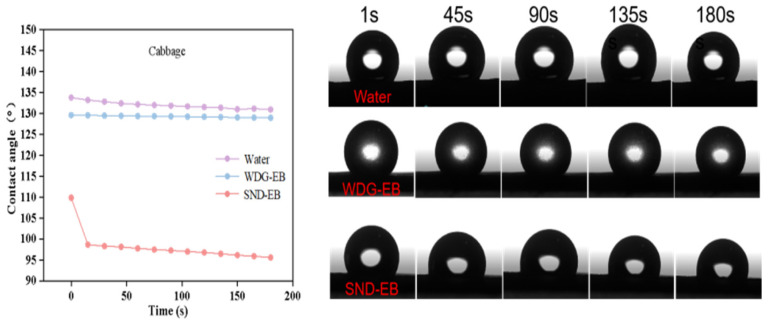
Dynamic contact angles of water, WDG-EB, and SND-EB on cabbage leaves.

**Figure 8:**
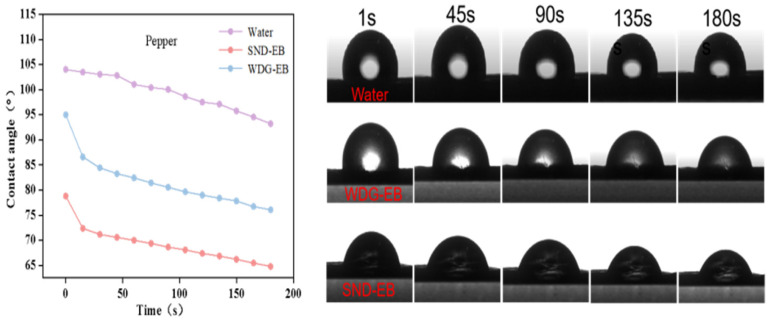
Dynamic contact angles of water, WDG-EB, and SND-EB pepper leaves.

The dynamic contact angle can provide information on the dynamic response and behavior of droplets under different conditions. Hydrophobic crops make up the majority, and improving the wettability of pesticides on hydrophobic leaves is of great significance. As shown in Figure 7, it could be seen that the dynamic contact angle of WDG-EB was closer to that of pure water on hydrophobic leaves (cabbage), while the dynamic contact angle of SND-EB decreased from 110° to 95° over time. As time goes by, the smaller the dynamic contact angle, the stronger the wetting effect of pesticide droplets. As shown in Figure 8, it could be seen that the dynamic contact angle of hydrophilic leaves (chili peppers) decreased continuously over time. After 180 s, the final stable contact angles of water, WDG-EB, and SND-EB were 93°, 76°, and 64°, respectively. It is obvious that SND-EB has better wetting properties than WDG-EB on hydrophilic leaves.

As is well known, solid nano-dispersions have superior wetting and permeability properties [45]. PEG 4000 has good water solubility and dispersibility. The diluted solution of SND-EB was sprayed onto the leaves, and the nanoparticles encapsulated in PEG 4000 quickly dispersed, which can lead to an imbalance in interfacial tension and displacement of the contact line [46]. The thin nanofluid wedge-shaped region was formed near the three-phase contact line, which restricts the nanoparticles within the wedge-shaped height. This promotes the diffusion of nanofluids and reduces the contact angle.

The diluted pesticide droplets will cause a high-speed impact on the surface of crops during the process of pesticide spraying in the field. Some droplets may splash and rebound, causing the active ingredient to be unable to reach the target crop, resulting in losses and reducing the utilization rate of pesticides.

**Figure 9:**
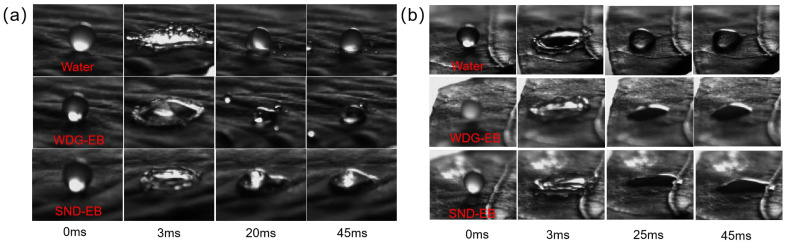
(**a**) The bouncing and wetting effects of water, WDG-EB, and SND-EB on cabbage leaves; (**b**) the bouncing and wetting effects of water, WDG-EB, and SND-EB on pepper leaves.

This study simulated the dynamic wetting process of diluted solution of SND-EB, WDG-EB, and pure water on hydrophilic leaves (chili pepper) and hydrophobic leaves (cabbage). As shown in Figure 9a, all droplets reached their maximum propagation at around 3 ms, rebounded to their maximum height at 20 ms, and reached a stable state at around 45 ms. Pure water and the WDG-EB solution showed significant splashing and rupture on cabbage leaves, and the droplets underwent significant retraction at the final stable state. However, SND-EB droplets did not rebound or splash significantly, and it was clearly observed that the droplets continued to diffuse, eventually completely wetting the leaf surface. As shown in Figure 9b, all droplets did not splash or rupture on the pepper leaves, which exhibited different wettability. At a stable state of 45 ms, SND-EB had a larger wetting area compared to the other two types of droplets. This may be related to the carrier PEG 4000. When PEG 4000 is dissolved in water, the molecules form a hydration layer in the solution, which can prevent water loss and maintain the stability of the solution. The PEG chain contains a large number of oxygen atoms, hydroxyl groups, and other hydrophilic groups, which can form hydrogen bonds with water and promote electrostatic attraction to the negatively charged leaf surface, thereby improving wettability.

**Figure 10:**
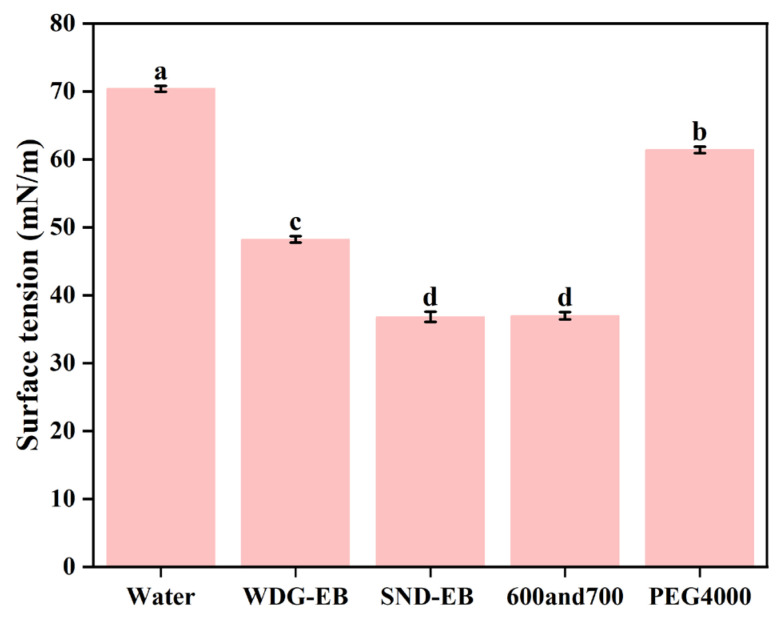
Surface tension of water, WDG-EB, and SND-EB. Different letters in the plot indicate a significant difference at *p* < 0.05.

The splashing, rupture, or rebound of droplets can cause pesticides to miss their targets during spraying, resulting in losses. The prepared SND-EB can minimize the splashing loss during drug spraying and improve the effective utilization rate of the pesticide. Surface tension was measured using a surface tension meter. The surface tension decreases with the increase in solution concentration. The smaller the surface tension, the better the spreading and wetting effect of the pesticide solution, which helps to penetrate the active ingredients and improve pesticide utilization efficiency. It was found that the surface tension of water was 70.39 mN/m, as shown in Figure 10. The surface tension of SND-EB was 36.79 mN/m, while the surface tension of the commercial WDGs was 48.19 mN/m. The surface tension of the mixture of emulsifiers 600 and 700 was 36.95 mN/m, and the surface tension of PEG 4000 was 61.3 mN/m. The reason for the low contact angle and surface tension of SND-EB may be that emulsifier 600 and emulsifier 700 exhibited good dispersion and wetting effects. 

**Figure 11:**
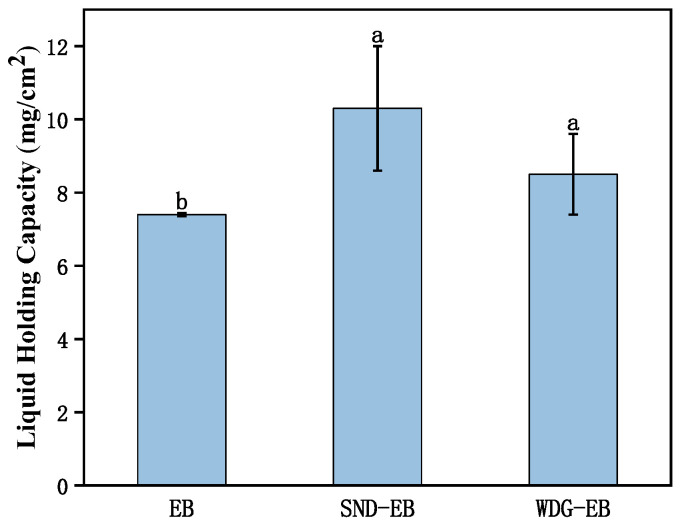
Leaf retention of water, WDG-EB, and SND-EB. Different letters in the plot indicate a significant difference at *p* < 0.05.

The retention affinity of solution on leaves is the key to evaluating the effective utilization rate of pesticides. As shown in Figure 11, the leaf retention of SND-EB was 10 mg/cm^2^, which was greater than that of commercially available WDG-EB and EB, indicating that SND-EB had a stronger ability to remain on the leaf surface. This result was consistent with the previous results of contact angle and surface tension. This can improve the utilization rate of pesticides, extend the prevention and control time, and reduce repeated application.

It is not difficult to see that SND-EB has excellent blade adhesion and wettability through contact angle measurement, bounce experiment, surface tension, and retention measurement. The small particle size and large specific surface area of SND-EB increase the coverage of pesticides on targets. Solid dispersion technology changes the crystal form of pesticide, causing SND-EB to transition from qualitative to amorphous, resulting in higher Gibbs free energy and increased solubility to achieve solubilization. The auxiliary effects of both the carrier and surfactant further enhance the wettability and adhesion of SND-EB.

### 3.5. Sustained-Release Behavior

**Figure 12:**
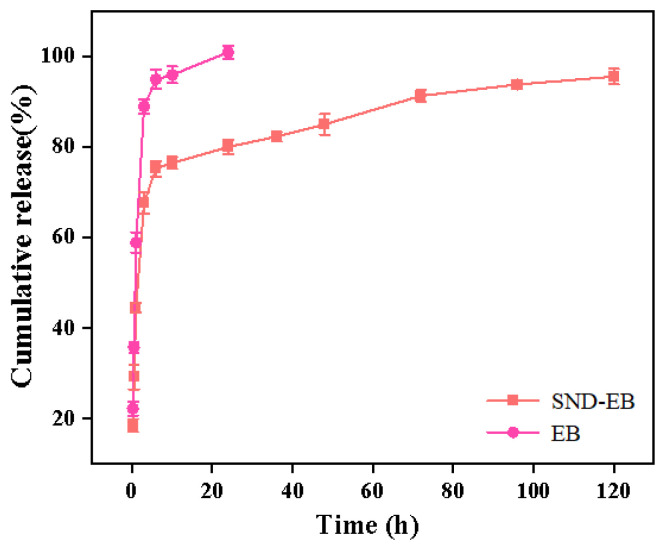
Sustained-release curves of EB and SND-EB.

As shown in Figure 12, the release rate of EB was very fast, and the cumulative release amount reached 90% within 6 h, and the active substance was completely released within 24 h. The release curve of SND-EB exhibited a biphasic pattern, characterized by rapid release followed by slow and sustained release. In the release medium, when the concentration of pesticides in the medium was below the equilibrium value, the active ingredients dissolved or separated from the active adsorption sites, migrated to the surface of the nanoparticles, and diffused into the medium. Therefore, the permeability of drug ingredients and the interaction between active ingredients play a crucial role in the release of pesticides. Low permeability and interaction between active ingredients can lead to slower release rates [47,48]. The active substance on the surface of SND-EB was rapidly released during the initial period. The SND-EB was continuously released within 120 h. PEG 4000 was used as a carrier for the preparation of sustained-release formulations in the pharmaceutical field, indicating its excellent sustained-release performance. The interaction between PEG 4000 and EB may be the reason for the slow and sustained release of the drug.

By fitting the cumulative release rate and release time of the drug, Mt represents the cumulative release rate of the drug at a certain moment, and t represents the release time. The results (from Table 1) showed that the release heights of SND-EB and EB followed first-order kinetics. Korsmeyer–Peppas analysis revealed that the n values of SND-EB and EB were 0.47 and 0.7, respectively, between 0.45 and 0.89. The Higuchi model has a low R^2^ of only about 0.6, indicating that both EB and SND-EB release processes are dominated by diffusion but accompanied by slight interference from dissolution or swelling during the release process. The initial diffusion of drugs through matrix pores briefly conforms to the Higuchi model, while in the middle and later stages, the matrix absorbs water and swells slightly, the diffusion path is prolonged, and the release rate decreases.

### 3.6. Insecticidal Activity Against Spodoptera Exigua

**Figure 13:**
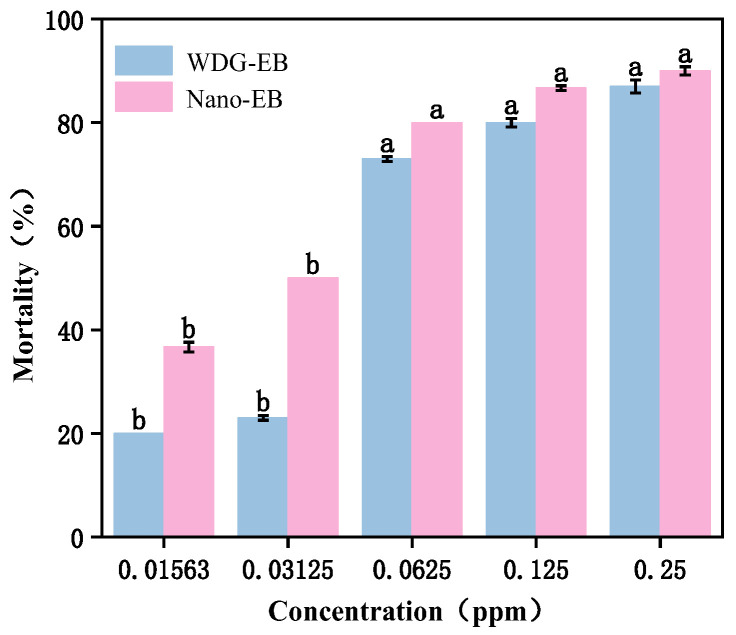
Insecticidal activities of SND-EB and WDG-EB against *S. exigua*. Different letters in the plot indicate a significant difference at *p* < 0.05.

*Spodoptera exigua* (*S. exiga*) is a polyphagous insect that feeds on various crops. Abamectin benzoate is considered the most suitable candidate for inhibiting *S. exiga* outbreaks due to its safety for mammals [49,50,51]. Therefore, beet armyworm was selected as a model organism to evaluate the biosafety of SND-EB and WDG-EB. From Figure 13, it can be seen that the mortality rate of beet armyworm continued to rise with increasing concentration, and the insecticidal effect of SND-EB was significantly higher than that of WDG-EB. From Table 2, it can be seen that the LC_50_ of SND-EB and WDG-EB were 0.026 and 0.049 mg/L, respectively. The toxicity of SND-EB to beet armyworm was 1.8 times that of WDG-EB. The results showed that the insecticidal effect of SND-EB was superior to that of commercially available WDG-EB. SND-EB had good wetting and dispersing properties, which can better penetrate crop leaves and thus improve the killing effect on target pests. SND-EB was composed of small-sized nanoparticles that can enter insect bodies through contact, inhalation, or ingestion, improving efficacy by increasing permeability and the retention adhesion effect [52,53,54].

### 3.7. Cytotoxicity

**Figure 14:**
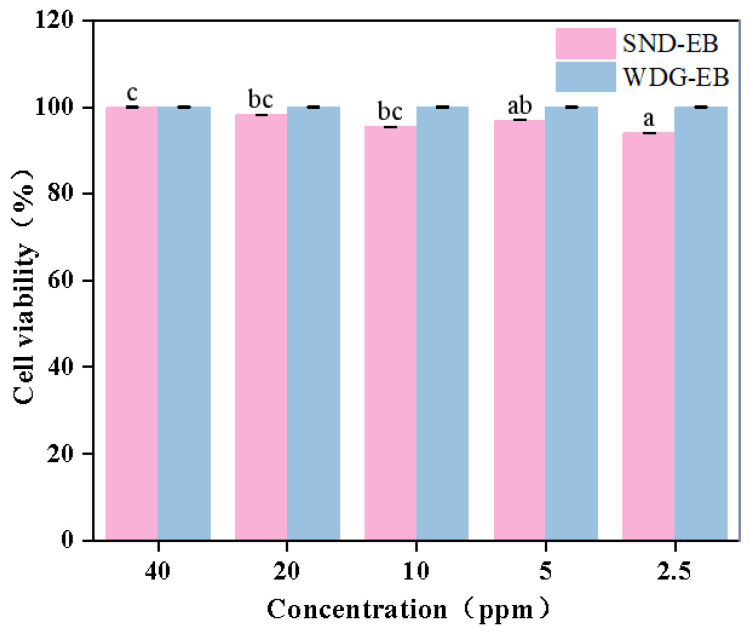
Cell viability of SND-EB and WDG-EB. Different letters in the plot indicate a significant difference at *p* < 0.05.

The safety of pesticides is crucial in the process of pesticide application. Cytotoxicity evaluation is one of the important methods for evaluating pesticide safety. The survival rate and metabolic characteristics of HepG2 cells can be used to evaluate the cytotoxicity of SND-EB [55]. As shown in Figure 14, the cell viability of WDG-EB remained unchanged with the change in concentration gradient, while the cell viability of SND-EB slightly changes but still remains at a high level (cell viability greater than 90%). The results indicated that the prepared SND-EB had weak cytotoxicity against HepG2 cells and good biocompatibility.

## 4. Conclusions

Herein, a 50% content of SND-EB with 17 nm was developed using PEG 4000 as a carrier by the melting method. The nanoscale size of SND-EB was conducive to the adsorption of surfactants on the surface of nanoparticles, resulting in excellent dispersibility and stability of the solid dispersion system. Under the action of the carrier and surfactant, SND-EB exhibited strong leaf-wetting and high leaf-holding capacity, and showed excellent dynamic wetting behavior in the simulated leaf bounce experiment. The retention performance further confirmed that the small size effect of nanoparticles endowed SND-EB with excellent leaf sedimentation ability and efficient pesticide transport efficiency. The SND-EB had a sustained-release effect that could effectively reduce pesticide loss in plants and decrease pesticide dosage and toxicity, thereby improving pesticide utilization and reducing environmental pollution. In addition, the control effect of SND-EB on *S. exigua* was 1.8 times higher than that of commercial WDGs, demonstrating excellent insecticidal efficacy. SND-EB emphasized the potential of solid nano-dispersion preparation technology in developing effective and environmentally friendly pesticide formulations with excellent wetting and adhesion behavior, spreading abilities, and strong insecticidal capabilities. The development of SND-EB provides a powerful reference for the development of novel nano-formulations of poorly soluble pesticides with great application potential in the control of pests and diseases in future agriculture.

## Figures and Tables

**Table 1:** Mathematical models corresponding to pesticide release from various formulations.

Sample	Boxlucas1 Equation	R^2^
SND-EB	$t=\frac{10}{7}\ln\left(\frac{84.68}{84.68-Mt}\right)$	0.924
EB	$t=\frac{1}{0.93}\ln\left(\frac{96.84}{96.84-Mt}\right)$	0.994

**Table 2:** Insecticidal activities of SND-EB and WDG-EB against *S. exigua*.

Formulation	Toxicity Regression Equation	LC_50_ (mg/L)	95% Confidence Limit	R^2^
SND-EB	Y = 2.394 + 1.510X	0.026	0.06–0.047	0.938
WDG-EB	Y = 2.417 + 1.851X	0.049	0.026–0.083	0.904

## Data Availability

Data is contained within the article.
